# *Thunbergia laurifolia* Exhibits Antifibrotic Effects in Human Hepatic Stellate Cells

**DOI:** 10.1155/2017/3508569

**Published:** 2017-12-13

**Authors:** Ratchadaporn Namsen, Noppamas Rojanasthien, Seewaboon Sireeratawong, Piyanuch Rojsanga, Wutigri Nimlamool, Saranyapin Potikanond

**Affiliations:** ^1^Department of Pharmacology, Faculty of Medicine, Chiang Mai University, Chiang Mai, Thailand; ^2^Graduate School, Chiang Mai University, Chiang Mai, Thailand; ^3^Department of Pharmaceutical Chemistry, Faculty of Pharmacy, Mahidol University, Bangkok, Thailand

## Abstract

Leaves of* Thunbergia laurifolia *(TL) have been reported to have antioxidation, anti-inflammatory, detoxifying, and hepatoprotective effects. However, studies relating to antifibrotic activity have not been reported. Currently, there is no standard treatment for hepatic fibrosis. This study aimed to investigate the antifibrotic activity of TL in human hepatic stellate LX-2 cells. Results from cell viability and cell death assays showed that the extract at high concentrations was toxic to LX-2 cells. TL extract reversed the transformation of LX-2 cells to myofibroblast-like characteristics in response to stimulation by transforming growth factor-beta 1. This action may be associated with the effect of TL in suppressing *α*-SMA and collagen-I production observed by immunofluorescence study and western blot analysis. Additionally, TL extract significantly decreased MMP-9 activity which is consistent with the reduction of* MMP-9*,* MMP-2*, and* TIMP-1 *gene expression. The effect of TL in suppressing fibrosis may be associated with its ability to inhibit the activation of p38 MAPK and Erk1/2 kinases as examined by western blot analysis. Our study provides convincing evidence that TL possesses antifibrotic activity which may be through the suppression of TGF-β_1_-mediated production of MMPs, collagen-1, and *α*-SMA in hepatic stellate cells.

## 1. Introduction

A consequence of chronic liver inflammation that leads to the activation of hepatic stellate cells (HSCs) is a major cause of hepatic fibrosis [1, 2]. One of the unique characteristics of the activated HSCs is a remarkable increase in extracellular matrix components including *α*-smooth muscle actin (*α*-SMA) and collagen [3]. Hepatic fibrosis can eventually cause liver cirrhosis, which can be identified by the abnormal structure of the liver where there are fibrous scar and nodule formation of hepatocytes [4]. The number of cirrhosis related death of the world's population was reported to be over a million in 2010 [5].

HSCs are unique mesenchymal cells that account for about 5–8% of the cells in the liver [6]. Normally, HSCs are in a quiescent stage. However, liver injury or chronic inflammation results in HSC activation and differentiation into myofibroblast-like cells. These cells have high expression level of *α*-SMA which has been proved to be a marker in development and progression of fibrosis [7]. *α*-SMA plays a major role in fibroblast contractility in the process of wound healing and tissue fibrosis [8]. The excess of *α*-SMA production can be found upon transforming growth factor-beta 1 (TGF-*β*_1_) stimulation [9]. TGF-*β*_1_ is secreted from activated HSCs and known to be an essential mediator of fibrogenesis [10, 11]. It plays crucial roles in many biological processes including cell growth, differentiation, apoptosis, and ECM production [10–12]. Additionally, human fibroblast can be stimulated to produce more MMP-9 upon TGF-*β*_1_ exposure [13]. MMPs are proteases which promote degradation of ECM components. MMP-9 or gelatinase B is known to play a major role in remodeling the ECM by digesting many molecules, including collagen-I [13]. Besides MMPs, tissue inhibitors of metalloproteinases (TIMPs) can also affect the outcome of fibrosis [14].

Several signal transduction pathways are responsible for contributing to liver fibrosis. Mitogen-activated protein kinases (MAPK), especially Erk1/2, are involved in proliferation and activation of HSCs as well as in regulation of many cellular functions, including cell growth and apoptosis, which can lead to a worsening of hepatic fibrosis [15, 16]. Moreover, the activation of p38 MAP kinase is also involved in HSC activation and transformation [17].

To the best of our knowledge, there is no standard treatment for hepatic fibrosis. Therefore, searching for novel agents with antifibrotic properties is our main focus. Plants are important sources and have been used as medicines for a long time. One of those is* Thunbergia laurifolia* (TL) or commonly known as blue trumpet vine or laurel clock vine [18]. The major constituents of TL include phenolic compounds, flavonoids, caffeic acid, and rosmarinic acid. TL has been reported to exhibit antioxidant, anti-inflammatory, detoxifying, and hepatoprotective effects [19–23]. However, investigation relating to antifibrotic activity in human HSCs has not been documented yet. Therefore, this study aimed to investigate whether TL extract affects the level of *α*-SMA, collagen-I, and MMPs in human HSCs. The possible molecular signal transduction pathways in which TL may interfere were also examined.

## 2. Materials and Methods

### 2.1. Preparation of* Thunbergia laurifolia* Extract

*Thunbergia laurifolia *(TL) extract was prepared by the Ouay-Un-Osoth Co., Ltd. Briefly, the leaves of TL (herbarium number PYTL2013) were washed and dried at 40°C in a hot-air oven. Fifty kilograms of dried TL leaves was pulverized into coarse powder. TL powder was boiled in 500 liters of distilled water at 100°C by reflux method twice, each for 2 h. The filtrate was evaporated and spray-dried at input temperature of 185°C (temperature of the heater) and then at output temperature of 86°C (temperature of the extract). Crude extract was dark brown and slightly bitter. Crude extract could be dissolved freely in water but slightly in 95% ethanol. The water extract of TL was evaluated to contain total phenolic content (18.51 ± 0.10 grams of gallic acid equivalent per 100 grams of dried extract), total flavonoid content (3.29 ± 0.10 grams of quercetin equivalent per 100 grams dried extract), caffeic acid (0.14 ± 0.02), and rosmarinic acid (0.24 ± 0.04) measured by thin layer chromatography.

### 2.2. Cell Culture

Human hepatic stellate cell line LX-2 (Merck, Darmstadt, Germany) was used in this study. Cells were cultured in Dulbecco's modified Eagle' medium (DMEM; Gibco), supplemented with 2% fetal bovine serum (FBS), 2 mM L-glutamine, 100 U/mL penicillin, and 100 *μ*g/mL streptomycin and incubated at 37°C 5% CO_2_.

### 2.3. Cell Viability Assay

LX-2 cells were cultured in 96-well plates at a density of 1 × 10^4^ cells/well in DMEM with 2% FBS for 24 h. Cells were treated with various concentrations of TL extract. After 24 h, cell viability was performed using MTT (3-(4,5-dimethylthiazolyl-2)-2,5-diphenyltetrazolium bromide). The change in color intensity was measured at the absorbance of 570 nm by a microplate reader (BioTek Instruments, USA).

### 2.4. Trypan Blue Exclusion Assay

Trypan blue exclusion test was performed for evaluating the percentage of cell death. HSCs were plated in 24-well plates at a density of 3 × 10^4^ cells/well and cultured in DMEM supplemented with 2% FBS for 24 h at 37°C 5% CO_2_. Then, 0.5 mg/mL of* Thunbergia laurifolia* crude extract was added to each well and cells were incubated for 0, 6, 9, 12, and 24 h at 37°C, 5% CO_2_. Cells were trypsinized and diluted in 0.4 % trypan blue dye at a 1 : 1 dilution and counted by using a haemocytometer.

### 2.5. Gelatinase Zymography

Cells were seeded in 3 cm dishes at a density of 0.3 × 10^6^ cells per well and cultured for 24 h. After TL treatment for 24 h, culture supernatants were collected and mixed with nonreducing sample buffer, and samples were separated by SDS-PAGE in a cold running condition. Following electrophoresis, the gels were washed twice in 2.5% Triton X-100 for 30 min at RT. The gels were then incubated with substrate buffer (50 mM, Tris-HCL, and 10 mM CaCl_2_ pH 8) overnight. The gels were stained with 0.5% Coomassie Blue R250 in 50% methanol and 10% glacial acetic acid for 30 min, and then destaining was performed. The intensity of each band was evaluated using ImageJ software.

### 2.6. Quantitative RT-PCR

LX-2 cells were seeded at a density of 0.3 × 10^6^ cells/well in 3 cm dishes. Cells were stimulated with or without TGF-beta 1 (1 ng/mL) for 24 h before further incubated with different concentrations of TL extract for 24 h at 37°C 5% CO_2_. Total RNA was extracted by a total RNA extraction kit (Vivantis, USA) according to the manufacturer's protocol. cDNA was prepared using reverse transcriptase master mix (Toyobo, Japan) following the ReverTra Ace® qPCR RT master mix protocol. Real-time PCR reaction was performed (2 min at 50°C and then 2 min at 95°C for activation, 15 s at 95°C, 15 s at 58°C, and 1 min at 72°C for 40 cycles of amplification) using SYBR® Green PCR Master Mix (Applied Biosystems, USA). A set of primers included TIMP-1: L: CTTCTGCAATTCCGACCTCGT, R: CCCTAAGGCTTGGAACCCTTT; MMP-2: L: ACATCAAGGGCATTCAGGAG, R: GCCTCCGTATACCGCATCAAT; MMP-9: L: CCCGGAGTGAGTTGAACCA, R: GGATTTACATGGCACTGCCA; and GAPDH: L: ATGGGGAAGGTGAAGGTCG, R: GGGGTCATTGATGGCAACAATA.

### 2.7. Western Blot

LX-2 cell lysates were prepared by adding 1x reducing Laemmli buffer into the sample dishes. Samples were collected, heated at 95°C for 5 min, separated by SDS-PAGE, and electroblotted onto PVDF membrane (GE Healthcare Life Sciences, Germany). Membranes were blocked with 5% skim milk in TBS-T (0.02 M Tris-HCl, pH 7.6, 0.0137 M NaCl, and 0.1% Tween 20) at RT for 1 h. Membranes were then incubated with an appropriate antibody (Cell Signaling Technology, USA) at 4°C overnight. Primary antibodies used included a 1 : 1,000 dilution of an anti-*α*-SMA antibody, or an anti-collagen-I antibody, or an anti-Akt (Ser 473) (D9E) antibody, a phosphospecific anti-Erk1/2 (Thr 202/Tyr 204) antibody, or a phosphospecific anti-p38 MAPK, and a 1 : 10,000 dilution of an anti-beta-actin antibody. After three washes with TBS-T, membranes were incubated with a 1 : 5000 dilution of an appropriate horseradish peroxidase-conjugated secondary antibody (KPL, USA) for 2 h, at RT. Immune complexes were detected using enhanced chemiluminescence reagent. The intensity of immunoreactive bands was analyzed and quantified using ImageJ software.

## 3. Results

### 3.1. The Effects of TL on LX-2 Cell Viability and Cell Death

**Figure 1:**
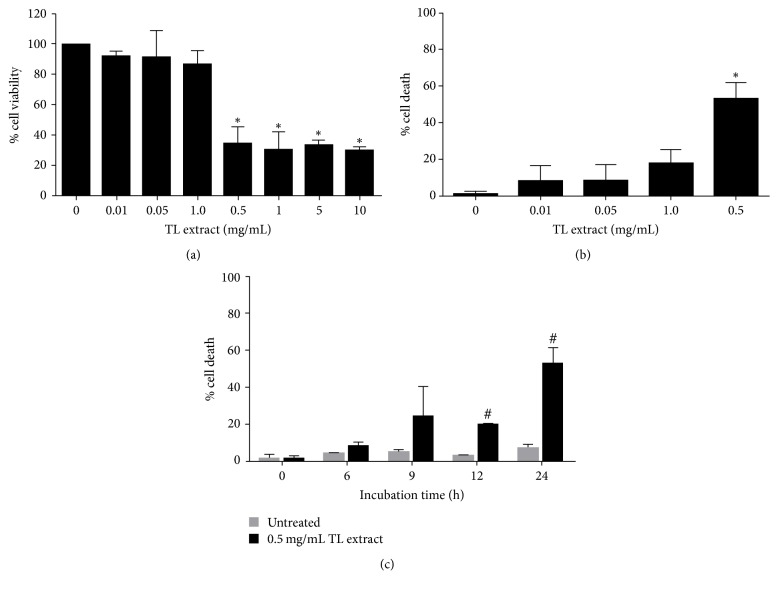
Effects of TL extract on cell viability and cell death. Bar chart indicates the percent of cell viability of LX-2 cells treated with TL extract (0–10 mg/mL) for 24 h and cell viability was measured by MTT assay (a). Cell death was measured by trypan blue exclusion test of cells treated with different concentrations of TL extract (b) and at different time points of cells treated with TL at 0.5 mg/mL (c). ^*∗*^*P* < 0.05 indicates statistical significance from untreated group. ^#^*P* < 0.05 indicates statistical significance from time point 0 of 0.5 mg/mL TL extract.

Since we focused mainly on antifibrotic activity of TL, we first evaluated its cytotoxicity to human hepatic stellate cell line (LX-2) by MTT assay. We found that cells treated with TL extract at a range of concentrations (0.01, 0.05, 0.1, 0.5, 1, 5, and 10 mg/mL) for 24 h showed significant reduction in cell viability, especially at 0.5, 1, 5, and 10 mg/mL as shown in Figure 1(a). The IC50 of TL extract was 0.2 mg/mL. Since results from cell viability test showed that TL extract at high concentrations exhibited a drastic decrease in cell viability, we speculated that the reduction of cell viability was due to induction of cell death. Therefore, we performed trypan blue exclusion assay and found that TL extract induced LX-2 cell death in a concentration-dependent manner. The percent cell death was 8.12 ± 8.35%, 8.49 ± 8.60%, 17.88 ± 8.49%, and 53.16 ± 8.63% for cells treated with TL extract at 0.01, 0.05, 0.1, and 0.5 mg/mL, respectively (Figure 1(b)). We next examined the cytotoxic effect of TL at different time points and discovered that TL extract tended to induce cell death at early time point (6 h) and the percentage of cell death gradually increased over time (Figure 1(c)). Data showed that TL extract induced approximately 53% of cell death after 24 h of incubation, whereas the control (untreated) group showed no observable cell death at all time points. Based on our observation that TL extract at concentrations between 0.01 and 0.1 mg/mL did not have any cytotoxic effect to LX-2 cells, we therefore used these nontoxic concentrations to further explore antifibrotic activity of TL.

### 3.2. The Effects of TL on Reversing TGF-*β*_1_-Induced Cell Transformation

**Figure 2:**
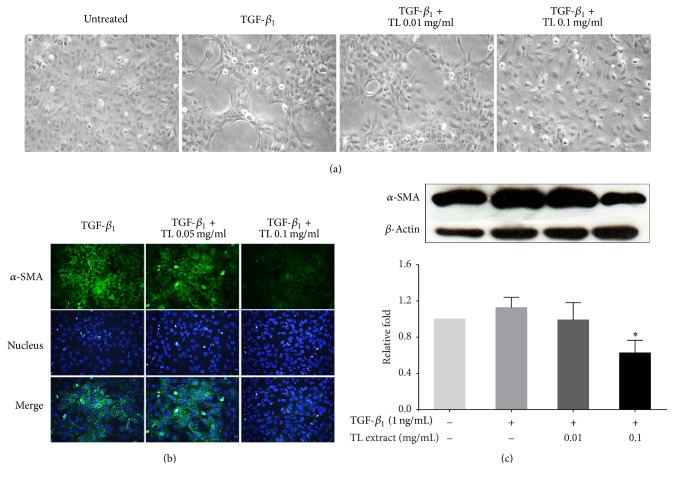
Effects of TL extract on reversing the morphology of TGF-*β*_1_-treated cells. Morphology of LX-2 cells observed by a phase-contrast microscope (a). Immunofluorescence study of *α*-SMA (b), and western blot analysis of *α*-SMA (c) in LX-2 cells stimulated with or without TGF-*β*_1_ and treated with TL extract. Actin was used as a loading control. ^*∗*^*P* < 0.05 indicates statistical significance from TGF-*β*_1_-treated group.

A known cytokine, TGF-*β*_1_, is normally produced during liver injury. This cytokine can stimulate hepatic stellate cells to increase the production of extracellular matrix (ECM) [24] and *α*-smooth muscle actin (*α*-SMA) [9, 25]. Upon TGF-*β*_1_ stimulation, LX-2 cells can be activated and transformed into myofibroblasts and lose their original characteristics [26].  Figure 2(a) shows that LX-2 cells without any treatment exhibited normal cell morphology showing star-shaped stellate cells with discrete cell-to-cell contact. In contrast, LX-2 cells stimulated with TGF-*β*_1_ induced cell transformation to myofibroblast-like morphology. These cells exhibited cell stretching and clumping with many obvious cell-free areas. Interestingly, TGF-*β*_1_-stimulated cells treated with TL extract (0.01 mg/mL and 0.1 mg/mL) reversed transformation of LX-2 cell to myofibroblast in a concentration-dependent manner.

### 3.3. The Effect of TL on Suppressing *α*-SMA and Collagen Production

**Figure 3:**
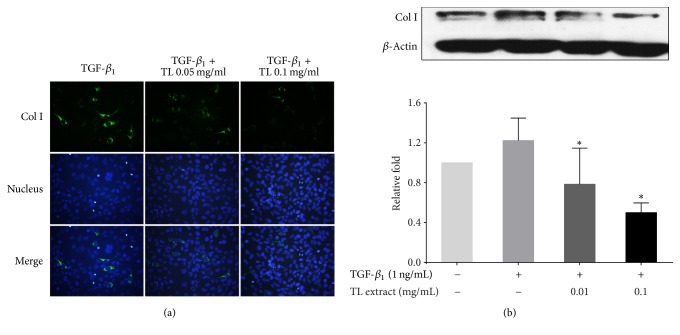
Effects of TL extract on collagen-I expression. Immunofluorescence study of collagen-I (a) and western blot analysis of collagen-I (b) in LX-2 cells stimulated with or without TGF-*β*1 and treated with TL extract. Actin was used as a loading control. ^*∗*^*P* < 0.05 indicates statistical significance from TGF-*β*_1_-treated group.

Since TGF-*β*_1_-induced transformation has been reported to be associated with an increase in *α*-SMA content [9, 27, 28], we therefore performed immunofluorescence study to evaluate *α*-SMA production. We observed that treating cells with TGF-*β*_1_ potently induced *α*-SMA protein production in the cytoplasm. Interestingly, TL extract at 0.05 and 0.1 mg/mL could drastically reduce *α*-SMA production (Figure 2(b)). The blue signal from Hoechst 33342 staining for nuclear detection showed approximately equal number of cells on glass cover slips, and the nuclear signal intensity was similar in all groups. Consistent with the results from immunofluorescence study, western blot analysis detecting immunoreactive bands of *α*-SMA revealed that TL extract could significantly reduce the production of *α*-SMA upon TGF-*β*_1_ stimulation (Figure 2(c)). The maximal reduction was observed to be approximately 0.63 ± 0.14-fold compared to that of the TGF-*β*_1_-stimulated group. We also determined whether the expression of important molecules involved in modulating extracellular matrix is suppressed by the effect of TL extract. The results from immunofluorescence study for collagen-I were similar to those observed in *α*-SMA staining. When the concentration of TL extract was increased, collagen-I protein production was significantly reduced (Figure 3(a)). Results from western blot confirmed the observation from immunofluorescence where a concentration-dependent reduction of collagen-I was observed. TGF-*β*_1_-stimulated cells treated with 0.01 and 0.1 mg/mL of TL extract showed the reduction of collagen-I production (0.78 ± 0.36-fold and 0.5 ± 0.1-fold), respectively, compared to the collagen-I level in TGF-*β*_1_-stimulated cells without TL extract exposure which was 1.22 ± 0.22-fold.

### 3.4. The Effect of TL on Inhibiting MMP-9 Activity

**Figure 4:**
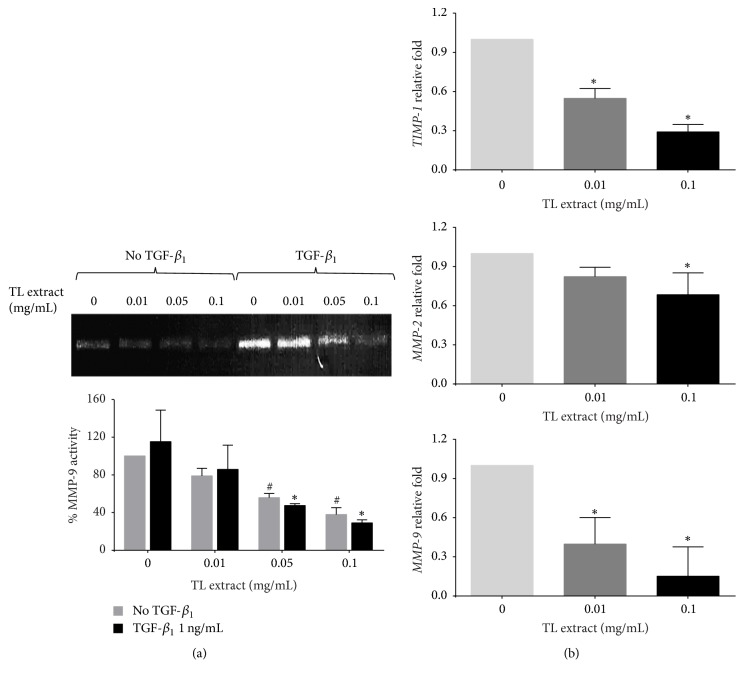
Effects of TL extract on MMP-9 activity and mRNA expression of* TIMP-1*,* MMP-2*, and* MMP-9* genes. MMP-9 activity of LX-2 cells were investigated by gelatin zymography. Bar graphs in zymogram represent the percentage of MMP-9 activity (a). ^#^*P* < 0.05 indicates statistical significance from no TGF-*β*_1_ group. ^*∗*^*P* < 0.05 indicates statistical significance from TGF-*β*_1_-treated group. Gene expression of* TIMP-1*,* MMP-2*, and* MMP-9* was measured by quantitative RT-PCR (b). GAPDH was used as an internal control. ^*∗*^*P* < 0.05 indicates statistical significance from TGF-*β*_1_-treated group.

Based on the fact that MMP-9 (gelatinase B) is one of fibrotic markers reported to be upregulated in liver fibrosis and is a major MMP in basement membrane-like ECM remodeling [29], we therefore determined whether TL extract can suppress MMP-9 activity. Data from zymographic analysis showed that the extract at noncytotoxic concentrations (0.01, 0.05, and 0.1 mg/mL) could effectively reduce MMP-9 activity (Figure 4(a)). The percentage of MMP-9 activity of cells treated with TL extract at 0.01, 0.05, and 0.1 mg/mL was reduced to 78.89 ± 8.04%, 55.77 ± 4.47%, and 37.94 ± 7.24%, respectively. Moreover, even though cells were stimulated with TGF-*β*_1_, TL extract was still able to suppress MMP-9 activity in a concentration-dependent manner. The percent reduction of MMP-9 activity was 85.76 ± 25.79%, 47.46 ± 1.93%, and 29.06 ± 3.24% for cells treated with TL extract at 0.01, 0.05, and 0.1 mg/mL, respectively. We further explored the effect of TL on suppressing MMP-9 gene expression along with other relevant molecules including MMP-2 and TIMP-1. Data from quantitative PCR confirmed that the extract could be able to suppress these genes in a concentration-dependent manner (Figure 4(b)).

### 3.5. The Effects of TL on Intracellular Signaling Pathways

**Figure 5:**
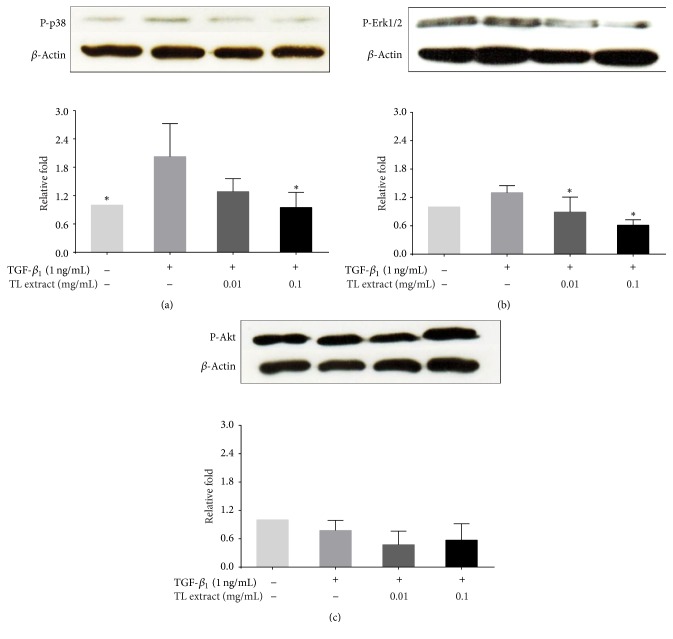
Effects of TL extract on phosphorylation of p38, Erk1/2, and Akt in LX-2 cells induced by TGF-*β*_1_ and treated with TL extract. Phosphorylation of p38 MAPK, Erk1/2, and Akt is shown in (a), (b), and (c), respectively. The bar graphs represent the relative fold of phosphorylated p38, Erk1/2, and Akt kinases. ^*∗*^*P* < 0.05 indicates statistical significance from TGF-*β*_1_-treated group.

Several signaling molecules are involved in fibrogenic process in response to TGF-*β*_1_ stimulation. These molecules include p38 MAPK and Erk1/2 proteins [15, 17]. Thus we defined the mechanism of action whether TL suppresses fibrosis via inhibiting the activation of these molecules. We found that TGF-*β*_1_ alone increased the activation of p38 MAPK protein. On the other hand, TGF-*β*_1_-stimulated cells treated with TL extract showed a significant decrease in phosphorylation of p38 MAPK in a concentration-dependent manner (Figure 5(a)). Similarly, phosphorylation of Erk1/2 was reduced when the concentration of TL extract was increased (Figure 5(b)). However, we observed no change in the phosphorylation status of Akt kinase which is a crucial molecular marker for survival signaling that plays a crucial role in the balance of HSC activation and apoptosis (Figure 5(c)).

## 4. Discussion

Hepatic fibrosis is a wound-healing response to chronic liver injury characterized by progressive inflammation and deposition of extracellular matrix (ECM) components [30]. During the development of fibrosis, hepatic stellate cells (HSCs) or myofibroblast-like cells are activated to synthesize excessive collagen and *α*-SMA leading to liver cirrhosis [4, 31]. Currently, there is no standard treatment for liver fibrosis. Therefore, agents that can be able to inhibit the production of *α*-SMA and collagen in activated HSCs would be excellent candidates for developing a potential treatment for liver fibrosis.

Our study aimed to investigate the antifibrotic activity of* Thunbergia laurifolia*. We focused mainly on the plant's ability to suppress *α*-SMA and collagen production in LX-2 cells which are an activated human hepatic stellate cell line [26]. In order to examine the antifibrotic activity of TL, we first assayed for its cytotoxicity to LX-2 cells. Based on the data from cell viability test showing that the low range of concentrations did not show any toxicity to the cell, therefore, in this study we chose the nontoxic concentrations of TL (0.01–0.1 mg/mL) to evaluate the antifibrotic activity of TL.

It has been well known that TGF-*β*_1_ which is a key regulator of extracellular matrix (ECM) assembly and remodeling is involved in hepatic fibrosis [10, 11]. In general, TGF-*β*_1_ induces the expression of ECM proteins in mesenchymal cells and stimulates the production of protease inhibitors that prevent enzymatic breakdown of the ECM. Based on this reason, we used TGF-*β*_1_ to stimulate LX-2 cells for enhancing the production of *α*-SMA protein, collagen-I, and metalloproteinase-9 (MMP-9) and explored the inhibitory effects of TL on the level of *α*-SMA protein, collagen-I, and MMP-9 in TGF-*β*_1_-stimulated LX-2 cells. The results from phase-contrast microscopy showed that TL extract at 0.1 mg/mL could reverse the morphology of TGF-*β*_1_-stimulated LX-2 cells from star-like morphology to normal characteristics when compared to the untreated cells. The results suggest that there must be some changes in the level of specific cytoskeletal proteins in TL-treated cells. Surprisingly, TL extract significantly reduced the production of *α*-SMA. For *α*-SMA, when HSCs are activated, intense cytoplasmic *α*-SMA level is increased. This protein is an actin isoform and a specific marker for muscle cell differentiation [32]. The level of *α*-SMA production can be used to identify activated HSCs which exhibit a myofibroblastic phenotype [33–35]. Therefore, our finding that TL could potently suppress *α*-SMA production suggests the role of TL in inhibiting cell differentiation and transformation in response to TGF-*β*_1_ stimulation. Besides, TL also suppressed the production of collagen-I in TGF-*β*_1_-stimulated cells in a concentration-dependent manner. The data suggest that TL interferes with TGF-*β*_1_-signaling that normally activates HSCs to undergo morphological changes to a myofibroblastic phenotype.

Collagen generation and degradation mediated by matrix metalloproteinases and tissue inhibitors of metalloproteinases can affect the outcome of fibrosis [36]. Therefore, the balance between MMPs and TIMPs is important in collagen degradation. When we performed the experiment for measuring the activity of MMP-9 by gelatin zymography, we found that TL extract inhibited the production of MMP-9 in LX-2 cells. Additionally, even though LX-2 cells were stimulated with TGF-*β*_1_, TL extract was still able to significantly suppress MMP-9 activity in a concentration-dependent manner. When we performed quantitative RT-PCR for* MMP-2*,* MMP-9*, and* TIMP-1 *genes, significant decreases in the expression level of these genes were observed. It has been reported that the expression of MMPs and TIMPs is increased in liver fibrosis and contributes to both the progression and regression of fibrosis [2]. In particular, there are studies showing the correlation between the level of TIMP-1 in the serum and fibrosis activity [37], and this specific protein is now a component of noninvasive serum markers of fibrosis [38]. In short, our discovery that TL extract significantly reduced the expression of MMPs and TIMP-1 provides accumulated evidence. These data may be beneficial for further investigation to verify whether TL possesses a potent effect slowing the progression of hepatic fibrosis.

Several molecular signal transduction pathways are responsible for the pathogenesis of hepatic fibrosis. One of those signaling pathways is mitogen-activated protein kinase (MAPK) involved in proliferation, activation of HSCs, and regulation of many cellular functions, including apoptosis which can lead to a worsening of hepatic fibrosis [15, 17]. TGF-*β*_1_ stimulates the activation of p38 MAPK and Erk1/2 which further activate specific transcription factors for specific responsive elements of profibrotic genes including collagen-I. We, therefore, tried to define the possible molecular mechanism of action to explain how TL works in modulating TGF-*β*_1_-induced fibrosis. The results showed that TL extract could suppress phosphorylation of p38 MAPK and Erk1/2 which are involved in proliferation and activation of HSCs. The observation indicates that the activation of these two kinases is potently suppressed by the action of TL. We observed that TL extract did not suppress phosphorylation of Akt kinase. This indicates that Akt is still active in maintaining the balance of cell survival. This observation was consistent with the results from MTT assay showing that TL extract at the selected concentrations for examining antifibrotic activity did not interfere with the viability of LX-2 cells and, thus, TL extract does not have any effect on suppressing cell survival.

In conclusion, the present study demonstrated that TL possesses antifibrotic properties. The extract from this plant could reduce the production of fibrotic markers which include *α*-SMA, collagen-I, and MMPs. The possible mechanism of action of TL may be through its interference in TGF-*β*_1_-dependent signal transduction pathways: p38 MAPK and Erk1/2. Our finding provides for the first time that TL has potent ability to suppress the activation of hepatic stellate cells and suggests that TL could be a potential therapeutic agent for hepatic fibrosis.
